# Generational immigration status and gastric cancer incidence among Japanese descendants: an analysis of the Multiethnic Cohort (MEC) Study

**DOI:** 10.1007/s10552-026-02250-0

**Published:** 2026-09-24

**Authors:** Haejin In, Katherine De la Torre-Cisneros, Alexandra Adams, Chunxia Chen, Brijesh Rana, Lynne Wilkens, Meira Epplein

**Affiliations:** 1 Division of Surgical Oncology, Rutgers Cancer Institute, New Brunswick, NJ USA; 2 Department of Health, Behavior and Policy, Rutgers University, Piscataway, NJ USA; 3 Biostatistics Shared Resource, Rutgers Cancer Institute of New Jersey, New Brunswick, NJ USA; 4 University of Hawaiʻi Cancer Center, University of Hawaiʻi at Mānoa, Honolulu, USA; 5 Department of Population Health Sciences, Duke University, Durham, NC USA

**Keywords:** Stomach Neoplasm, Immigration, Japanese descendants, Generation

## Abstract

**Background:**

Racial and ethnic minorities in the United States experience disproportionately higher rates of gastric cancer (GC) compared to the general population. However, the impact of immigration history on these disparities remains unclear. We evaluated the association between immigration generation and GC risk among Japanese descendants.

**Methods:**

We analyzed data from the prospective, population-based Multiethnic Cohort (MEC) Study, which enrolled residents of Hawaii and California between 1993 and 1996. Participants who self-identified as Japanese or non-Hispanic White (NHW) were included. Cox proportional hazard models were used to examine the association between immigration generation of Japanese descendants and the risk of GC overall and by anatomical subtype, adjusting for demographic, dietary, and lifestyle variables.

**Results:**

Among 94,232 individuals (median age: 61 years; male 47.4%), 716 primary GC cases were identified. The risk of developing GC was higher among all three generations of Japanese descendants compared to NHWs. First generation had the highest risk (HR: 4.62, 95% CI 3.24–6.59), followed by the second generation (US-born, with both parents Japan-born; HR: 3.66, 95% CI 2.89–4.64; US-born, with one parent Japan-born, HR: 3.39, 95% CI 2.55–4.50), and the third generation (HR: 2.57, 95% CI 1.93–3.42). The association was more pronounced among non-cardia GC, with HRs of 5.89, 4.49, and 3.27 for the first, second, and third generations, respectively.

**Conclusions:**

GC risk declined across successive generations of Japanese descendants but remained significantly higher than in NHWs. Recognizing and monitoring these persistently high-risk populations may enable risk-stratified screening and inform mechanistic research into environmental, biological, and transgenerational drivers of GC susceptibility.

**Supplementary Information:**

The online version contains supplementary material available at https://doi.org/10.1007/s10552-026-02250-0.

## Introduction

Gastric cancer is the 5th most diagnosed cancer in the world and the 4th leading cause of cancer death worldwide [1]. The incidence of gastric cancer has wide geographic variation, with higher incidence in Asia and lower incidence in Western countries. In the United States, racial/ethnic variation in gastric cancer incidence is well described [2]. Gastric cancer incidence is markedly elevated in minorities, with roughly double the incidence rates in individuals of Asian (20.8), Black (18.4), and Hispanic (17.1) race/ethnicity compared to Non-Hispanic White (NHW) individuals (10.7 per 100,000 persons) [3]. The reason for these differences is an ongoing area of research, but thought to be due to several host and environmental factors that differ between minority and NHW populations. One hypothesis for the striking difference amongst races is that immigration status, and therefore environmental factors, may play a role in pathogenesis. For example, household crowding is much higher in immigrant populations when compared to NHW [4]. This could contribute to *H. pylori* transmission and even reinfection, with this bacterium thought to be one of the major drivers of gastric cancer development [5]. Further explanations include a difference in genetic predisposition, dietary patterns, and prevalence of smoking, all of which are thought to contribute to the varying rates of gastric cancer observed amongst US minorities [6–8].

Research on immigrants to the US from “high-risk” regions, mainly from Asia, has found that gastric cancer incidence declines with subsequent generations when compared to their native country [9]. Incidence amongst these immigrants nonetheless remains higher when compared to US NHW individuals, suggesting the compounding effect of environment and genetics. Prior studies have examined the independent effect of race and immigration on the development of gastric cancer [10–12]. However, these studies are largely based on cancer registry data and therefore could not capture individual-level exposures, generational history, or potential confounders necessary to examine these relationships comprehensively. Additionally, few studies have systematically investigated the combined effect of these factors within a prospectively followed cohort of Japanese descendants.

Using data from the Multiethnic Cohort (MEC) Study, a large prospectively collected data set unique for its large representation of minority populations, we sought to evaluate the impact of generational immigration status on the risk of gastric cancer among Japanese descendants.

## Methods

### Study population

The MEC Study is a prospective cohort study that enrolled residents of Hawaii and California from 1993 to 1996 [13]. Men and women aged 45–75 years from five main racial/ethnic groups were enrolled: White, Black, Japanese, Latino, and Native Hawaiian. Cohort members, comprising over 215,000 participants, completed a comprehensive questionnaire at the time of enrollment. The questionnaire encompassed demographic factors, diet, medications, occupation, physical activity, family history of cancer, vitamin supplement intake, and immigration history. Detailed information about the cohort has been described elsewhere [13]. We received a dataset comprising 201,064 participants after excluding individuals who did not belong to one of the five main ethnic groups, those with prior gastric cancer diagnosis as reported in the questionnaire or from the tumor registry, and those with inconsistent dates (*n* = 14,585). We restricted our analysis to individuals who self-identified their ethnic or racial background as being Japanese or White (*n* = 106,136). Individuals were classified as Japanese if they reported Japanese ancestry either exclusively or in combination with another racial or ethnic background. Individuals classified as White reported only White ancestry. Participants with missing information on birthplace for themselves, their mother, or their father were further excluded (*n* = 762). Among those who self-identified as Japanese, we additionally excluded individuals whose own or parent’s birthplace was outside the US or Japan (*n* = 6,105). Among participants eligible at baseline (*n* = 99,401), we excluded those with invalid dietary information (*n* = 3,725), missing baseline covariate data for education (*n* = 685), BMI (*n* = 397), or smoking status (*n* = 774), and those with less than one year of follow-up (*n* = 4). During follow-up, participants who were diagnosed with non-adenocarcinoma gastric malignancies (*n* = 125) were also excluded. The final analytic cohort included 94,232 participants. (Supplementary Fig. 1).

### Assessment of generation status and covariates

Generational immigration status was defined based on the birthplace of each participant and their parents. Japanese descendants were defined as individuals who self-reported any Japanese background on the baseline survey question that allowed participants to select multiple racial or ethnic backgrounds. Japanese generational immigration status was categorized into 4 groups of Japanese descendants: (1) Japan-born, (first generation, *n* = 4,136); (2) US-born, both parents Japan-born (second generation, *n* = 23,241); (3) US-born, one parent Japan-born (second generation, *n* = 8,615); (4) US-born, both parents US-born (third generation or more, *n* = 18,461). A fifth group, consisting of all US-born individuals who self-identified as only non-Hispanic Whites (NHW) (*n* = 39,779), served as the reference group.

Covariates were obtained from the baseline questionnaire and included age, sex, education, family history of gastric cancer, body mass index (BMI), physical activity, fruit and sodium consumption, alcohol consumption, smoking status, and personal history of diabetes mellitus, ulcers, and polyps of the intestines. Fruit consumption (grams per day) and sodium consumption (milligrams per day) were categorized into quartiles based on the distribution across the entire baseline cohort, with the lowest quartile serving as the reference group for both variables.

### Outcome ascertainment

Our dataset included incident cases of cancer identified through 2014 from two state-wide SEER registries: the Hawaii Tumor Registry and the California State Cancer Registry. The primary outcome was incident gastric cancer adenocarcinoma (ICD Tenth Revision C160 to C169), restricted to Site Recode ICD-O-3/WHO 2008 8140–8147, 8210–8211, 8260–8263, 8480–8481, 8490 and 8560. Gastric cancer anatomical subsites were classified as cardia (C16.0) and non-cardia (C16.1–C16.9).

### Statistical analysis

Baseline characteristics were examined as categorical variables and summarized using frequencies and percentages, except for age, which was reported as median and range. Cox proportional hazard models were utilized to determine hazard ratios (HR) and 95% confidence intervals (CI) for the association between Japanese generational immigration status and gastric cancer risk, using NHW as the reference group. We used age used as the time scale, as this approach inherently adjusts for the age effects and improves model fit in observational studies [14]. Time at entry was defined as the age at enrollment, and censoring time was defined as age at first primary gastric adenocarcinoma diagnosis, loss to follow-up, or end of follow-up, whichever occurred first. A test for nonproportionality based on Schoenfeld partial residuals was performed to assess the Cox proportional hazards assumption [15].Sex violated this assumption and was therefore included in the model as a stratification variable. The final model was additionally adjusted for education (< 12 grade, ≥ 12 grade), family history of gastric cancer in first-degree relative (yes, no), BMI (< 25, 25 to <30, ≥30), alcohol consumption (no drink or <1 drink per day, ≥1 drink per day), smoking status (never, former, current), fruit consumption (quartiles), history of diabetes (yes, no), history of ulcer (yes, no), and history of polyps (yes, no).

Linear trends were evaluated among Japanese-ancestry participants by modeling generational categories as an ordinal continuous variable (1 = first generation; 2 = second generation, both parents Japan-born; 3 = second generation, one parent Japan-born; 4 = third generation) in Cox regression models. Stratified analyses were performed by sex, family history of gastric cancer, sodium consumption, and smoking status. Effect modification of the association between Japanese generational status and gastric cancer risk in the stratified analyses was evaluated by including interaction terms between the generational status variable and each covariate in the Cox models, with statistical significance assessed by the Wald test of cross-product terms [16]. Additionally, we assessed the risk of gastric cancer among the second generation as a combined group, merging individuals who were US-born with either one or both parents Japanese-born. As a secondary analysis, we evaluated the association between generational status and gastric cancer by anatomical subsite. For each subsite-specific analysis, participants diagnosed with gastric cancer at other anatomical subsites were retained in the risk set and censored at their date of diagnosis. A cumulative probability plot was generated to depict the probability of gastric cancer development by age and generational status.

Sensitivity analyses were conducted by restricting the cohort to participants with more than two and five years of follow-up to minimize potential reverse causality and exclude subclinical or undiagnosed prevalent gastric cancer present at baseline enrollment. We also performed sensitivity analysis restricted to US-born individuals who self-identified as NHW and whose parents were born in the US or Europe, to avoid misclassification, since some participants who self-identified as NHW had parents born in Latin America or Asian countries other than Japan. Additionally, we restricted the analyses to individuals who reported their ethnic or racial background as exclusively Japanese. Finally, to evaluate potential anatomical subsite misclassification, we performed a sensitivity analysis restricting non-cardia gastric cancer strictly to specific anatomical subsites (ICD-10 C16.1–C16.7), assessing overlapping and unspecified subsites (C16.8 and C16.9) as a separate outcome category.

Two-sided p-values <0.05 were considered statistically significant. Statistical analysis was conducted using SAS software version 9.4 (SAS Institute, Cary, NC, USA).

## Results

Among 94,232 participants included in the study, 716 gastric cancer cases were identified during a median follow-up of 20.2 years (1,669,991 person-years). Median age at baseline was 61 years (range 44–78 years), and males accounted for 47.4% of the entire study population. Participant demographics by gastric cancer status are summarized in Table 1. Compared to participants who did not develop gastric cancer, those who did were older, more likely to be male, of Japanese ethnicity, have a family history of gastric cancer, more likely to have less than a 12th grade education, and more likely to have a BMI of less than 25. At baseline, individuals who later developed gastric cancer reported higher fruit and sodium consumption and were more likely to be current or former smokers. They also reported higher rates of diabetes, intestinal polyps, and gastric or duodenal ulcers compared to those who did not develop gastric cancer. No significant differences were observed for alcohol consumption or physical activity by gastric cancer status.

**Table 1:** Characteristics of Japanese descendants and non-hispanic whites MEC participants by gastric cancer status

Characteristic	Gastric cancer (*n* = 716)	No gastric cancer (*n* = 93,516)	P VALUE^B^
	No. (%)	No. (%)	
Age, median (range), y	68 (46–77)	61 (45–78)	<0.0001
Person-years	6983	1,663,007	
Sex			
Male	453 (63.3)	44,192 (47.3)	<0.0001
Female	263 (36.7)	49,324 (52.7)	
Self-reported race			
NHW^a^	103 (14.4)	39,676 (42.4)	<0.0001
Japanese	610 (85.6)	53,840 (57.6)	
US Generation			
Not US-born	48 (6.7)	4088 (4.4)	<0.0001
US-born; both parents Japan-born	370 (51.7)	22,871 (24.5)	
US-born; one parent Japan-born	99 (13.8)	8516 (9.1)	
US-born; both parents US-born	96 (13.4)	18,365 (19.6)	
White US-born; both parents US-born	69 (9.6)	32,038 (34.2)	
White US-born; one or both parents non US-born	34 (4.8)	7638 (8.2)	
First-degree family history of gastric cancer			
No	600 (83.8)	87,329 (93.4)	<0.0001
Yes	116 (16.2)	6187 (6.6)	
Education			
<12 grade	375 (52.4)	31,591 (33.8)	<0.0001
≥12 grade	341 (47.6)	61,925 (66.2)	
Body mass index (kg/m^2^)			
<25	429 (59.9)	50,154 (53.6)	0.0006
25-<30	228 (31.8)	32,121 (34.4)	
≥30	59 (8.3)	11,241 (12.0)	
Alcohol consumption (per day)			
0-<1 drink	570 (79.6)	75,461 (80.7)	0.464
≥1 drink	146 (20.4)	18,055 (19.3)	
Physical activity (per week)			
1 h or less	132 (18.4)	16,986 (18.2)	0.811
2 to 3 h	194 (27.1)	25,031 (26.8)	
4 h or more	371 (51.8)	50,231 (53.7)	
No information	19 (2.7)	1268 (1.3)	
Fruit consumption (grams per day, range)			
Quartile 1 (0–142.01)	169 (23.6)	23,703 (25.3)	0.0009
Quartile 2 (142.02–280.31)	165 (23.0)	24,699 (26.4)	
Quartile 3 (280.32–484.60)	183 (25.6)	24,839 (26.6)	
Quartile 4 (484.61–3545.6)	199 (27.8)	20,275 (21.7)	
Sodium consumption (mg per day, range)			
Quartile 1 (383.0–2271.9)	150 (21.0)	21,240 (22.7)	0.022
Quartile 2 (2272.0–3180.9)	174 (24.3)	25,806 (27.6)	
Quartile 3 (3181.0–4442.9)	204 (28.5)	25,846 (27.6)	
Quartile 4 (4443.0–16929.3)	188 (26.2)	20,624 (22.1)	
Smoking status			
Never	262 (36.6)	41,988 (44.9)	<0.0001
Former	339 (47.4)	38,496 (41.2)	
Current	115 (16.0)	13,032 (13.9)	
History of Diabetes			
No	633 (88.4)	85,433 (91.4)	0.005
Yes	83 (11.6)	8083 (8.6)	
History of ulcers			
No	589 (82.3)	82,665 (88.4)	<0.0001
Yes	127 (17.7)	10,851 (11.6)	
History of polyps of intestine			
No	646 (90.2)	86,344 (92.3)	0.035
Yes	70 (9.8)	7172 (7.7)	

*US* United States, mg milligrams

^a^ NHW is defined as those who were born in the US and self-identified as non-Hispanic White, regardless of where their parents were born

^b^ p-value was calculated excluding the no information category

Among Japanese descendants, 7.6% were non-US-born, 42.7% were US-born with both parents born in Japan-born, 15.8% were US-born with one parent born in Japan, and 33.9% were US-born with both parents born in the US. Sodium consumption was highest for the first generation and decreased with each subsequent generation, whereas BMI was lowest in the first generation and progressively increased across generations. First-generation immigrants were more likely to consume alcohol, have higher sodium intake, and be never-smokers compared with later generations of Japanese descendants (Supplementary Table 1).

Japan-born participants had a 4.62-fold higher hazard of developing gastric cancer compared to NHW (HR: 4.62, 95% CI 3.24–6.59). Among US born participants, those with both parents born in Japan had a 3.66-fold higher hazard (HR: 3.66, 95% CI 2.89–4.64), those with one parent born in Japan had 3.39-fold higher hazard (HR: 3.39, 95% CI 2.55–4.50), and those with both parents born in the US had a 2.57-fold higher hazard (HR: 2.57, 95% CI 1.93–3.42) compared to NHW. These findings indicate a significant linear decline in risk across successive generations (p-trend <0.0001) (Table 2). Sex-stratified analyses showed similar trends among both men and women (Table 2).

**Table 2:** Hazard ratios of gastric cancer incidence by generation status among Japanese descendants

Population	NHW^a^	First generation	Second generation, Both parents Japan-born	Second generation, one parent Japan-born	Third generation	p-trend
All subjects						
No. participants	39,779	4136	23,241	8615	18,461	
GC cases	103	48	370	99	96	
HR (95% CI)^b^	Reference	5.06 (3.58–7.14)	3.97 (3.17–4.96)	3.59 (2.72–4.73)	2.61 (1.97–3.47)	<0.0001
HR (95% CI)^c^	Reference	4.62 (3.24–6.59)	3.66 (2.89–4.64)	3.39 (2.55–4.50)	2.57 (1.93–3.42)	<0.0001
Men						
No. participants	18,725	1209	11,648	4227	8836	
GC cases	65	25	237	67	59	
HR (95% CI)^b^	Reference	6.44 (4.06–10.23)	4.02 (3.03–5.32)	3.81 (2.71–5.36)	2.46 (1.72–3.52)	<0.0001
HR (95% CI)^c^	Reference	5.73 (3.57–9.19)	3.50 (2.60–4.70)	3.42 (2.41–4.85)	2.31 (1.61–3.32)	<0.0001
Women						
No. participants	21,054	2904	11,593	4388	9625	
GC cases	38	23	133	32	37	
HR (95% CI)^b^	Reference	4.02 (2.40–6.76)	3.89 (2.69–5.62)	3.21 (2.00–5.14)	2.91 (1.84–4.62)	<0.0001
HR (95% CI)^c^	Reference	3.82 (2.20–6.62)	3.82 (2.54–5.74)	3.24 (1.98–5.31)	3.02 (1.88–4.87)	<0.0001

*NHW* non-hispanic whites, *GC* gastric cancer

^a^ NHW is defined as those who were born in the US and self-identified as non-Hispanic White, regardless of where their parents were born

^b^ Model 1 adjusted for age

^c^ Model 2 adjusted for age, sex, family history, sodium and fruit consumption, alcohol intake, education, body mass index, history of diabetes, ulcers, polyps, smoking

**Fig. 1:**
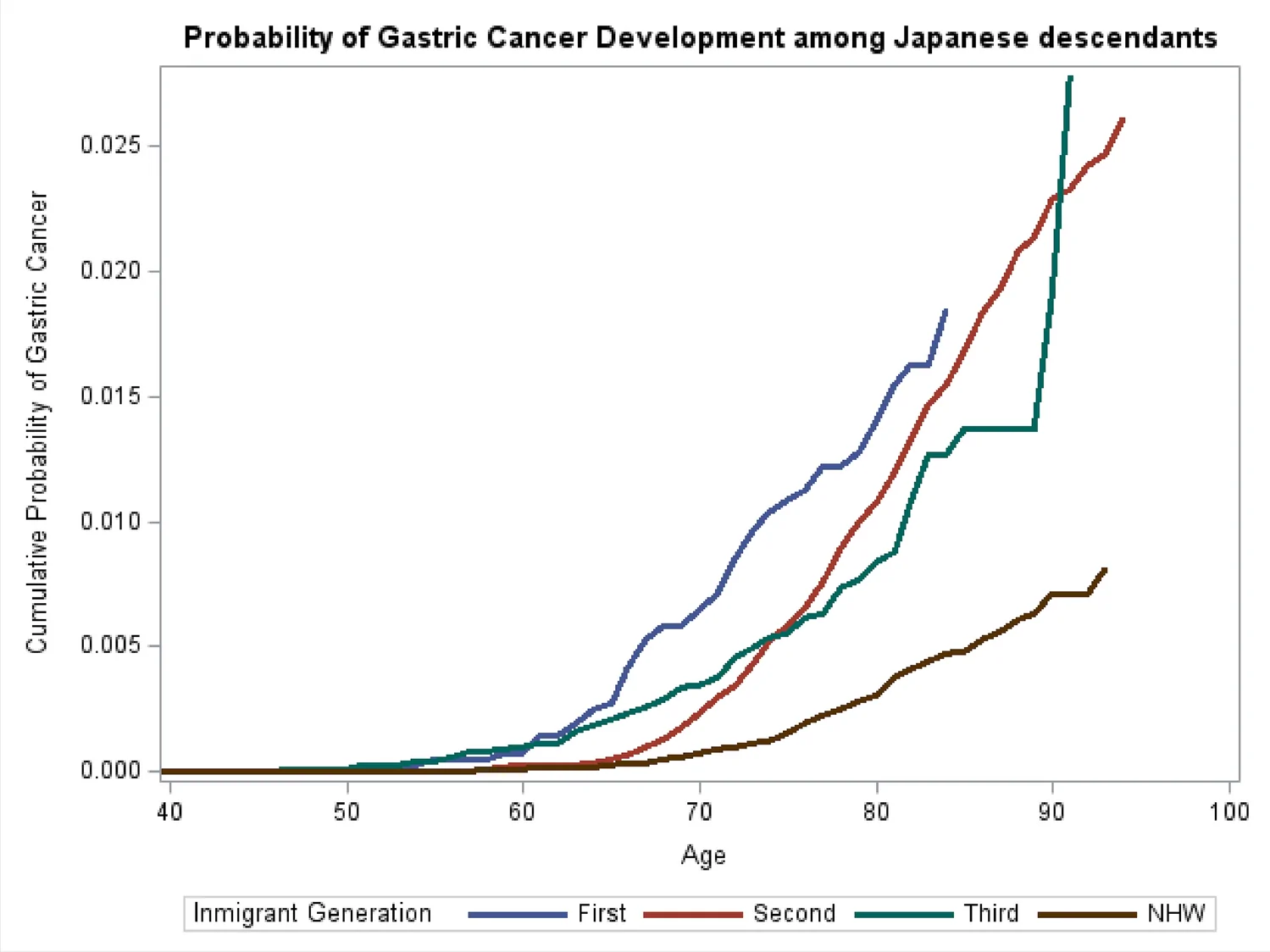
Probability of gastric cancer development among Japanese descendants

When second-generation Japanese descendants were analyzed as a single group, irrespective of whether one or both parents were born in Japan, the risk of gastric cancer was 3.59 times higher than for NHW individuals (HR: 3.59, 95% CI 2.85–4.51; p-trend<0.0001) (Supplementary Table 2), supporting a progressive attenuation of risk across generations rather than a marked decline limited to the transition from the first to second generation. Family history of GC, education, BMI, smoking status, and sodium intake did not modify the association between Japanese generational status and GC risk (Supplementary Table 3). Across the lifespan, individuals of Japanese ethnicity consistently demonstrated a higher cumulative probability of developing gastric cancer beginning at 50 years old compared to NHW (log-rank p <0.0001) (Fig. 1).

The association between generational status and gastric cancer risk was driven predominantly by non-cardia gastric cancer. Compared with NHWs, first-generation Japanese descendants had a nearly 6-fold increased risk of non-cardia cancer (HR: 5.89, 95% CI 3.96–8.77); second-generation descendants had a 4.5-fold increased risk (HR: 4.49, 95% CI 3.40–5.92); and third-generation descendants had an approximately 3-fold increased risk (HR: 3.27, 95% CI 2.33–4.59) (Table 3). In sensitivity analyses restricting non-cardia cancer to specific subsites (C16.1–C16.7), associations strengthened across all generations: first-generation HR = 7.13 (95% CI: 4.53–11.23), second-generation HR = 4.97 (95% CI: 3.57–6.12), and third-generation HR = 3.45 (95% CI: 2.30–5.16). Overlapping and unspecified subsites (C16.8–C16.9) showed weaker, though still elevated, associations (Supplementary Table 4). In contrast, associations between generational status and cardia cancer risk were weaker, with no statistically significant linear trend across generations (p-trend = 0.219). The risk of gastric cancer by anatomical subsite differed significantly across all generational groups (p-value for overall comparison = 0.016) (Table 3).

**Table 3:** Hazard ratios of cardia and non-cardia gastric cancer by generation immigration status among Japanese descendants

	Total population	NHW^a^	First generation	Second generation	Third generation	p-trend
Cardia						
No. participants	94,232	39,742	4136	31,856	18,461	
GC cases	121	37	5	60	19	
HR (95% CI)^b^		Reference	1.87 (0.72–4.85)	1.82 (1.16–2.87)	1.31 (0.74–2.34)	0.219
Non-cardia						
No. participants	94,232	39,742	4136	31,856	18,461	
GC cases	595	66	43	409	77	
HR (95% CI)^b^		Reference	5.89 (3.9–8.77)	4.49 (3.40–5.92)	3.27 (2.33–4.59)	<0.0001
P for heterogeneity between groups			0.026	0.001	0.020	
P for heterogeneity for overall comparison between groups: 0.016						

*GC* gastric cancer

^a^ NHW is defined as those who were born in the US and self-identified as non-Hispanic White, regardless of where their parents were born

^b^ Adjusted for age, sex, family history, sodium and fruit consumption, alcohol intake, education, body mass index, history of diabetes, ulcers, polyps, smoking

In sensitivity analysis, the associations between generational immigration status and gastric cancer risk were consistent across multiple specifications. Results were similar when the reference group was restricted to self-identified NHW individuals born in the United States with both parents born in the United States or Europe, as well as when participants with the first two or five years of follow-up were excluded. Additionally, analyses restricted to individuals reporting Japanese ancestry only yielded substantively similar results to the main analyses (Supplementary Table 5).

## Discussion

In this study exploring generational immigration status among Japanese descendants and gastric cancer incidence, we found that first-, second- and third-generation Japanese descendants were all at increased risk of gastric cancer, particularly non-cardia gastric cancer, compared to NHW. The first generation had the highest risk, with gastric cancer incidence progressively declining across subsequent generations. However, risk remained significantly elevated in all generations compared with NHW individuals.

Our finding of differential risk by immigration generation in Japanese American individuals is supported by the landmark paper by Kamineni et al., who found that first generation Japanese immigrants to the US had higher incidence of gastric cancer when compared to US Whites, but lower incidence rates when compared to rates in Japan, and the incidence rates decreased with subsequent generations [10]. These findings are consistent with the progressive attenuation in gastric cancer risk observed across generations in our study. Kolonel et al., in a cross-sectional study including 1216 interviews conducted among Hawaiian Japanese and Caucasians adults over age 45, also found similar results, that Japanese immigrants to Hawaii (non-US born) had higher GC incidence rates than Japanese born in the US [17].

The relationship between immigration and *H. pylori* infection may partly explain the observed generational effect. Individuals migrating from countries with a high prevalence of *H. pylori* are more likely to acquire the infection during childhood, a period when chronic gastric inflammation can become established and influence long-term susceptibility to gastric carcinogenesis [18, 19]. Persistent childhood infection has also been associated with virulent H. pylori strains, such as cagA/vacAs1m1, which may disrupt early-life microbiota and immune regulation [20, 21]. Although there is evidence of intergenerational decline in H. pylori prevalence among immigrant populations; [22] the persistence elevation in gastric cancer risk among third-generation Japanese descendants suggests that *H. pylori* alone does not fully explain the observed associations and additional biological mechanisms may be involved.

Potential explanations include dietary patterns, microbiome composition, and epigenetic programming, which are factors that may be transferred through generations and interact with the post-immigration environment to influence the risk of gastric cancer development. Studies show that a Westernized diet and lifestyle alter the gut microbiome, causing US-associated microbiota to replace native Asian microbiota [23, 24].These effects become more pronounced with longer residence in the US [23].Second-generation Japanese descendants showed a preference for Japanese food, while third-generation Japanese descendants prefer Western foods [25]. Also, alterations in the microbiota in Japanese descendants have been suggested to induce insulin resistance, a factor that favors carcinogenesis, in non-diabetic Japanese men [23].Epigenetic modifications may also change in response to environmental exposures after migration. Differences in DNA methylation between Italian migrants and non-migrants were found in a cross-sectional study after adjusting for dietary and lifestyle factors [26]. However, epigenetic studies in immigrant populations can be complex due to research design, sample size, and data availability. Furthermore, there may be variations in the distribution of genetic factors, such as genetic polymorphisms in inflammatory pathways associated with gastric cancer, among immigrant generations that could shape the risk of gastric cancer towards some specific ethnicity. Among Japanese individuals, for instance, a particular polymorphism of Interleukin-1 (IL-1) genes has been reported to modify the risk of gastric cancer independently of *H. pylori* infection or smoking status [27, 28].The intricate relationship between race/ethnicity, migration history, and the biological causes of gastric cancer may become clearer with further research using multigenerational prospective studies, integrating multilevel data where molecular profiling may be particularly informative in unraveling gastric cancer mechanisms influenced by transgenerational ethnic differences.

Heterogeneity in gastric cancer risk by generational status between cardia and non-cardia subsites might reflect different etiologies and exposure patterns for each subtype, such as acculturation and lifestyle shifts, diet changes, or *H. pylori* prevalence [25, 29]. Non-cardia gastric cancer has been strongly related to *H. pylori*, high salt consumption, and low socio-economic status, whereas cardia gastric cancer has been shown to be related to obesity, gastroesophageal reflux disease (GERD), and high-fat diets [30, 31].This heterogeneity may reflect declining exposure to *H. pylori* and changing dietary patterns across successive generations [32, 33]. As immigrants adapt to new dietary patterns, they tend to shift towards consuming less salty and more processed and fatty foods [26, 34]. This change might reduce the risk of non-cardia gastric cancer while maintaining the same risk level for cardia gastric cancer, comparable to that of White individuals living in the U.S. Further research is needed to explore the potential of acculturation, lifestyle shifts, diet changes, and *H. pylori* prevalence on the risk of gastric cancer by anatomical subtype in relation to generational immigration.

In a previously published paper utilizing the MEC from our research team, we showed that some gastric cancer risk factors were specific to certain racial/ethnic groups [35]. Among Japanese descendants, these factors included a family history of gastric cancer, a lower education level, and current smoking. In the present study, we evaluated these factors, as well as sodium consumption and body mass index, and found no evidence that they modified the association between generational status and gastric cancer risk. This suggests that the association between generational status and gastric cancer risk is not fully explained by these established risk factors and may reflect distinct environmental, biological, or early-life influences. Notably, in sex-stratified analyses, although confidence intervals overlapped, the elevated risk observed among third-generation Japanese descendants appeared to be numerically higher among women than men. While this finding should be interpreted with caution, it is noteworthy given recent reports of increasing gastric cancer incidence among younger women in the US [36].

Beyond gastric cancer, these generational shifts align with broader migrant epidemiological studies demonstrating a dual trajectory in cancer risk [37, 38]. Across successive generations, infection-associated and origin-endemic cancers, such as liver and cervical cancers, consistently decline [39, 40], whereas hormone- and lifestyle-associated "Western" cancers, including breast, colorectal, and prostate cancers, progressively increase with acculturation [41, 42].

While population-based gastric cancer screening programs are routinely implemented in several high incidence countries, the disproportionate burden of gastric cancer among certain U.S. populations highlights the need for risk-based screening approaches domestically [43, 44]. The marked differences in gastric cancer risk across immigrant generations, even within the same racial/ethnic group, suggest that generational status could help identify individuals most likely to benefit from screening [45]. Several studies have demonstrated the cost-effectiveness of gastric cancer screening among high-risk groups in the U.S., including Asian descendants undergoing upper endoscopy [46], opportunistic upper endoscopy during colonoscopy [47, 48] and the use of non-invasive biomarkers such as pepsinogen [49]. Health systems may be able to increase the effectiveness and accessibility of gastric cancer surveillance programs by concentrating resources on the generations of immigrants who are most at risk, improving the cost-effectiveness of screening in certain racial/ethnic groups.

A major strength of this study is the large sample size of Japanese descendants, with self-reported information on parents’ place of birth and over two decades of follow-up. However, this study also has several limitations. First, self-reported race and ethnicity may be subject to misclassification, as these are social constructs that can be influenced by social desirability bias. Consequently, self-identification may not accurately reflect genetic ancestry, which can play an important role in the biological determinations of gastric cancer risk. Second, information on *H. pylori* infection, the primary cause of non-cardia gastric cancer, was not available. However, we were able to adjust for some correlated variables, such as history of gastritis and ulcers, which are often linked to *H. pylori* infection [50]. Although multiple potential confounders were considered, residual confounding by unmeasured factors, such as pre-cancerous lesions or autoimmune gastritis, may still be present. Third, the relatively small number of individuals within each immigrant generation in the subgroups analysis, particularly for cardia gastric cancer, may have limited the statistical power to detect significant associations. Finally, the MEC cohort was drawn from two specific regions, Hawaii and Los Angeles, which have long-established Japanese American communities and distinct migration and acculturation patterns; therefore, our findings may not be fully generalized to Japanese descendants residing in other US regions. Future efforts should focus on replicating these findings in geographically diverse cohorts that include detailed information on age at migration, selective migration, and duration of residence in the US. These factors, which are often unavailable, are critical for understanding sensitive periods during the life course that may influence exposure patterns and disease risk [51]. Such studies would help validate the observed generational patterns and assess their consistency across different populations and settings.

## Conclusion

Our study suggests that Japanese immigrants to the US, and their descendants, retain an elevated risk for gastric cancer, indicating complex etiologies in the natural history of gastric cancer related to race/ethnicity and geographical origin. A better understanding of the transgenerational risk determinants of gastric cancer in immigrants could inform targeted screening, early detection, and prevention programs. By accounting for the complexities of race/ethnicity’s interplay with generational immigration, healthcare systems can better respond to the disproportionate burden of gastric cancer in minority populations.

## Supplementary Information

Below is the link to the electronic supplementary material.

Supplementary file1 (PDF 265 KB)

## Data Availability

For information on how to gain access to the multiethnic cohort, please see: [**https://www.uhcancercenter.org/for-researchers/mec-data-sharing**] (https:/www.uhcancercenter.org/for-researchers/mec-data-sharing).
