# Supplementary material for: Generational immigration status and gastric cancer incidence among Japanese descendants: an analysis of the Multiethnic Cohort (MEC) Study
Source: Cancer Causes Control. 2026 Sep 24;37(10):168. doi: 10.1007/s10552-026-02250-0 (PMC13612697; doi:10.1007/s10552-026-02250-0)
Supplement: Supplementary file 1 [file 10552_2026_2250_MOESM1_ESM.pdf]

**Supplementary Material**

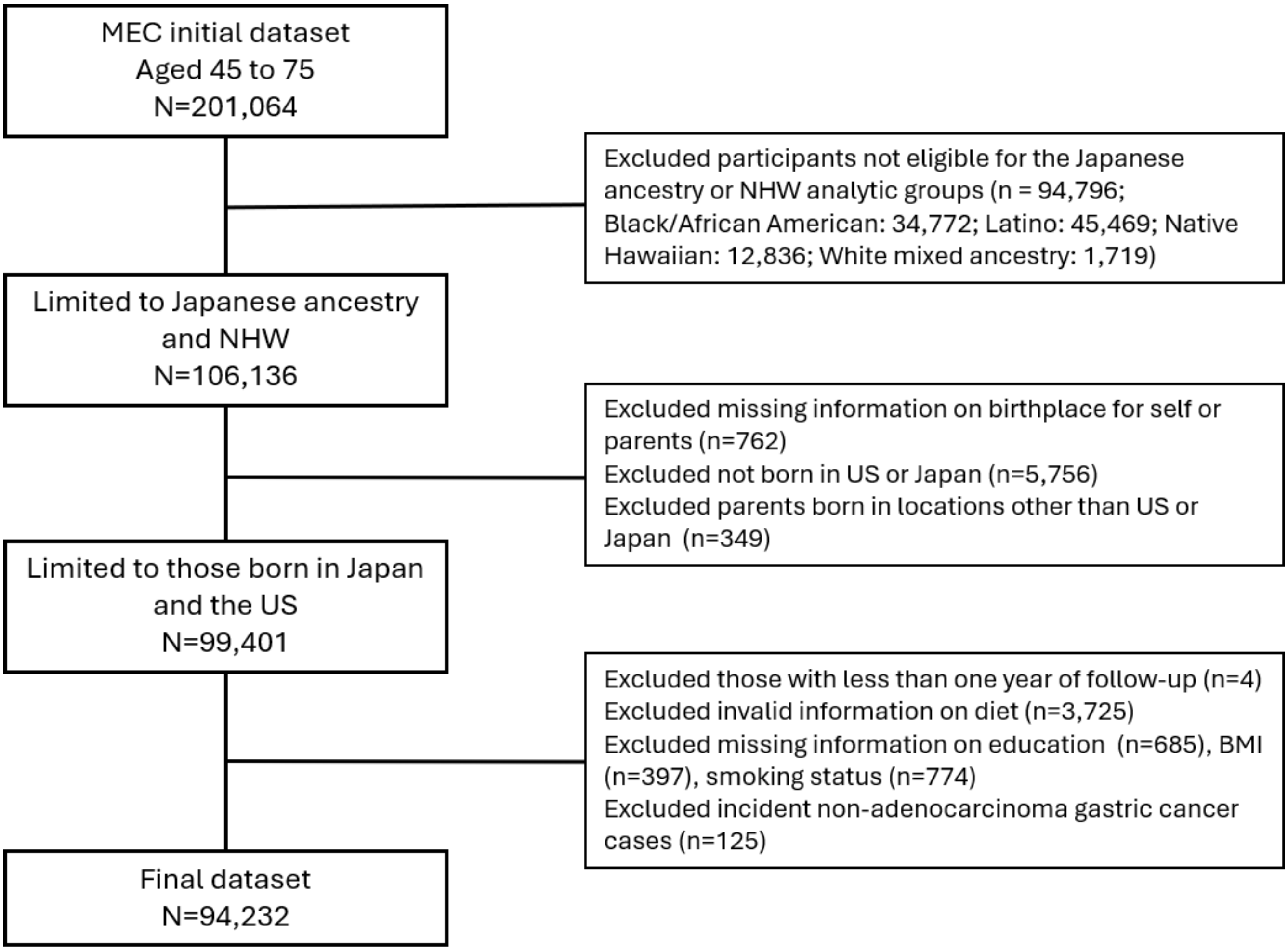


Supplementary Figure 1. Flowchart of participants' inclusion criteria

Supplementary Table 1. Characteristics of the MEC population by generational immigration status

| Characteristic | First generation (n=4136) | Second generation, both parents Japan-born (n=23241) | Second generation, one parent Japan-born (n=8615) | Third generation (n=18461) | NHW[a] (n=39779) | P Value |
|---|---|---|---|---|---|---|
| | No. (%) | No. (%) | No. (%) | No. (%) | No. (%) | |
| **Age, median (range), y** | 58 (45-77) | 69 (45-78) | 62 (45-77) | 52 (45-77) | 59 (45-78) | |
| **Person-years** | 77956 | 384699 | 155857 | 356706 | 694773 | |
| **Sex** | | | | | | |
| Male | 1209 (29.2) | 11648 (50.1) | 4227 (49.1) | 8836 (47.9) | 18725 (47.1) | <0.0001 |
| Female | 2927 (70.8) | 11593 (49.9) | 4388 (50.9) | 9625 (52.1) | 21054 (52.9) | |
| **Family History of gastric cancer** | | | | | | |
| No | 3757 (90.8) | 20410 (87.8) | 7768 (90.2) | 17348 (94.0) | 38646 (97.2) | <0.0001 |
| Yes | 379 (9.2) | 2831 (12.2) | 847 (9.8) | 1113 (6.0) | 1133 (2.8) | |
| **Education level** | | | | | | |
| <12 grade | 1754 (42.4) | 12828 (55.20) | 3291 (38.2) | 3871 (21.0) | 10222 (25.7) | <0.0001 |
| ≥12 grade | 2382 (57.6) | 10413 (44.8) | 5324 (61.8) | 14590 (79.0) | 29557 (74.3) | |
| **Body mass index (kg/m$^2$)** | | | | | | |
| <25 | 3183 (77.0) | 14959 (64.4) | 4996 (58.0) | 9230 (50.0) | 18215 (45.8) | <0.0001 |
| 25-<30 | 862 (20.8) | 7250 (31.2) | 2994 (34.7) | 6831 (37.0) | 14412 (36.2) | |
| >30 | 91 (2.2) | 1032 (4.4) | 625 (7.3) | 2400 (13.0) | 7152 (18.0) | |
| **Alcohol consumption (per day)** | | | | | | |
| 0-<1 drink | 3502 (84.7) | 20651 (88.9) | 7535 (87.5) | 15859 (85.9) | 28484 (71.6) | <0.0001 |
| ≥1 drink | 634 (15.3) | 2590 (11.1) | 1080 (12.5) | 2602 (14.1) | 11295 (28.4) | |
| **Physical activity (per week)** | | | | | | |
| 1 hour or less | 964 (23.3) | 4405 (18.9) | 1781 (20.7) | 3731 (20.2) | 6237 (15.7) | <0.0001 |
| 2 to 3 hours | 1021 (24.7) | 6119 (26.3) | 2327 (27.0) | 5491 (29.7) | 10267 (25.8) | |
| 4 hours or more | 2054 (49.6) | 12286 (52.9) | 4393 (51.0) | 9096 (49.3) | 22773 (57.2) | |
| No information | 97 (2.4) | 431 (1.9) | 114 (1.3) | 143 (0.8) | 502 (1.3) | |
| **Fruit consumption** | | | | | | |
| Quartile 1 | 1080 (26.1) | 4165 (17.9) | 2185 (25.3) | 6531 (35.4) | 9911 (24.9) | <0.0001 |
| Quartile 2 | 1093 (26.4) | 5536 (23.8) | 2269 (26.3) | 5021 (27.2) | 10942 (27.5) | |
| Quartile3 | 1073 (25.9) | 6894 (29.7) | 2232 (25.9) | 4037 (21.9) | 10786 (27.1) | |
| Quartile4 | 890 (21.5) | 6643 (28.6) | 1929 (22.4) | 2872 (15.6) | 8140 (20.5) | |
| **Sodium consumption** | | | | | | |
| Quartile 1 | 529 (12.8) | 5012 (21.6) | 1862 (21.6) | 3699 (20.0) | 10288 (25.8) | <0.0001 |
| Quartile 2 | 849 (20.5) | 6526 (28.1) | 2371 (27.5) | 5025 (27.2) | 11209 (28.2) | |
| Quartile 3 | 1220 (29.5) | 6574 (28.2) | 2473 (28.7) | 5239 (28.4) | 10544 (26.5) | |

| | | | | | | |
|---|---|---|---|---|---|---|
| Quartile 4 | 1538 (37.2) | 5129 (22.1) | 1909 (22.2) | 4498 (24.4) | 7738 (19.5) | |
| **Smoking status** | | | | | | |
| Never | 2283 (55.2) | 12072 (52.0) | 4186 (48.6) | 8513 (46.1) | 15196 (38.2) | <0.0001 |
| Former | 1249 (30.2) | 9252 (39.8) | 3374 (39.1) | 6976 (37.8) | 17984 (45.2) | |
| Current | 604 (14.6) | 1917 (8.3) | 1055 (12.3) | 2972 (16.1) | 6599 (16.6) | |
| **History of diabetes** | | | | | | |
| No | 3863 (93.4) | 20220 (87.0) | 7712 (89.5) | 16820 (91.1) | 37451 (94.1) | <0.0001 |
| Yes | 273 (6.6) | 3021 (13.0) | 903 (10.5) | 1641 (8.9) | 2328 (5.9) | |
| **History of ulcers** | | | | | | |
| No | 3680 (89.0) | 19914 (85.7) | 7509 (87.2) | 16689 (90.4) | 35462 (89.1) | <0.0001 |
| Yes | 456 (11.0) | 3327 (14.3) | 1106 (12.8) | 1772 (9.6) | 4317 (10.9) | |
| **History of polyps** | | | | | | |
| No | 3956 (95.7) | 20737 (89.2) | 7857 (91.2) | 17336 (93.9) | 37104 (93.3) | <0.0001 |
| Yes | 180 (4.3) | 2504 (10.8) | 758 (8.8) | 1125 (6.1) | 2675 (6.7) | |

Abbreviations: First generation: JA/JAJA; Japan-born, both parents Japanese-born; Second Generation: US/JAJA; US-born, both parents Japanese-born; Second Generation: US/JAUS; US-born, one parent Japanese-born; Third generation: US-born, both parents US-born

[a] NHW is defined as those who were born in the US and self-identified as White, regardless of where their parents were born

Supplementary Table 2. Hazard ratios of gastric cancer by generation status among Japanese descendants

| Population | NHW[a] | First Generation | Second generation | Third generation | p-trend |
|---|---|---|---|---|---|
| **All subjects** | | | | | |
| No. participants | 39779 | 4136 | 31856 | 18461 | |
| GC cases | 103 | 48 | 469 | 96 | |
| HR (95%CI)[b] | Reference | 5.06 (3.58-7.15) | 3.87 (3.12-4.81) | 2.62 (1.97-3.48) | <0.0001 |
| HR (95%CI)[c] | Reference | 4.62 (3.23-6.58) | 3.59 (2.85-4.51) | 2.57 (1.93-3.43) | <0.0001 |
| **Men** | | | | | |
| No. participants | 18725 | 1209 | 15875 | 8836 | |
| GC cases | 65 | 25 | 304 | 65 | |
| HR (95%CI)[b] | Reference | 6.45 (4.06-10.24) | 3.96 (3.02-5.20) | 2.46 (1.72-3.53) | <0.0001 |
| HR (95%CI)[c] | Reference | 5.73 (3.57-9.19) | 3.47 (2.61-4.62) | 2.31 (1.61-3.32) | <0.0001 |
| **Women** | | | | | |
| No. participants | 21054 | 2927 | 15981 | 9625 | |
| GC cases | 38 | 23 | 165 | 37 | |
| HR (95%CI)[b] | Reference | 4.03 (2.40-6.76) | 3.71 (2.59-5.32) | 2.93 (1.85-4.64) | <0.0001 |
| HR (95%CI)[c] | Reference | 3.80 (2.19-6.59) | 3.66 (2.46-5.43) | 3.03 (1.88-4.89) | <0.0001 |

Abbreviations: GC, gastric cancer

[a] NHW is defined as those who were born in the US and self-identified as White, regardless of where their parents were born.

[b] Model 1 adjusted for age

[c] Model 2 adjusted for age, sex, family history, sodium and fruit consumption, alcohol intake, education, body mass index, history of diabetes, ulcers, polyps, smoking

Supplementary Table 3. Subgroup analysis of gastric cancer risk by generation status, family history of gastric cancer, and smoking status.

| Characteristic | No. participants | GC cases | White race | First generation | Second generation, Both parent Japan-born | Second generation, one parent Japan-born | Third generation | Interaction term |
|---|---|---|---|---|---|---|---|---|
| | | | HR (95%CI) | HR (95%CI)[b] | HR (95%CI)[b] | HR (95%CI)[b] | HR (95%CI)[b] | |
| **Family history of GC** | | | | | | | | 0.264 |
| No | 87929 | 600 | Reference | 4.12 (2.77-6.14) | 3.60 (2.81-4.61) | 3.42 (2.54-4.62) | 2.61 (1.93-3.52) | |
| Yes | 6303 | 116 | Reference | 7.77 (2.70-22.36) | 4.35 (1.72-10.96) | 3.68 (1.33-10.13) | 2.71 (0.93-7.91) | |
| **Education** | | | | | | | | 0.525 |
| ≤12 grade | 31966 | 375 | Reference | 3.47 (1.97-6.10) | 3.70 (2.55-5.34) | 2.59 (1.64-4.11) | 2.84 (1.80-4.50) | |
| >12 grade | 62266 | 341 | Reference | 5.66 (3.58-8.95) | 3.50 (2.55-4.80) | 4.28 (2.98-6.15) | 2.47 (1.71-3.59) | |
| **Body mass index, kg/m²** | | | | | | | | 0.658 |
| <25 | 50583 | 427 | Reference | 5.30 (3.37-8.35) | 4.02 (2.84-5.70) | 3.85 (2.57-5.77) | 3.06 (2.01-4.67) | |
| 25-<30 | 32349 | 227 | Reference | 4.17 (2.06-8.43) | 3.22 (2.19-4.74) | 2.75 (1.70-4.46) | 2.33 (1.46-3.74) | |
| >30 | 11300 | 59 | Reference | 3.47 (0.46-26.03) | 3.92 (1.94-7.90) | 4.31 (1.94-9.59) | 1.82 (0.87-3.82) | |
| **Smoking status** | | | | | | | | 0.067 |
| Never | 42250 | 262 | Reference | 4.19 (2.31-7.61) | 3.12 (2.05-4.75) | 3.35 (2.05-5.47) | 3.20 (1.97-5.20) | |
| Former | 38835 | 339 | Reference | 4.05 (2.28-7.18) | 3.78 (2.72-5.26) | 3.06 (2.03-4.62) | 2.03 (1.31-3.12) | |
| Current | 13147 | 115 | Reference | 6.75 (3.20-14.22) | 4.46 (2.44-8.15) | 4.50 (2.28-8.87) | 3.05 (1.58-5.88) | |
| **Sodium consumption** | | | | | | | | 0.254 |
| Q1 | 21390 | 150 | Reference | 6.70 (2.89-15.52) | 4.64 (2.75-7.83) | 4.87 (2.64-8.99) | 3.38 (1.73-6.60) | |
| Q2 | 25980 | 174 | Reference | 5.52 (2.54-12.01) | 3.98 (2.49-6.38) | 2.90 (1.59-5.30) | 3.79 (2.22-6.46) | |
| Q3 | 26050 | 204 | Reference | 5.06 (2.77-9.24) | 2.65 (1.72-4.09) | 3.00 (1.81-4.97) | 1.70 (0.98-2.96) | |
| Q4 | 20812 | 188 | Reference | 2.81 (1.40-5.65) | 4.00 (2.46-6.51) | 3.19 (1.78-5.69) | 2.10 (1.18-3.71) | |

Abbreviations: GC, gastric cancer

[a] NHW is defined as those who were born in the US and self-identified as non-Hispanic White, regardless of where their parents were born.

[b] Model adjusted for age, sex, fruit consumption, alcohol intake, history of diabetes, ulcers, polyps, family history, education, body mass index, smoking, and sodium consumption. The variable tested for subgroup analysis was excluded from the model.

Supplementary Table 4. Hazard ratios of cardia and non-cardia gastric cancer by generation immigration status among Japanese descendants

| | Total Population | NHW[A] | First Generation | Second Generation | Third Generation | p-trend |
|---|---|---|---|---|---|---|
| **Cardia** | | | | | | |
| No. participants | 94232 | 39713 | 4093 | 31447 | 18384 | |
| GC cases | 121 | 37 | 5 | 60 | 19 | |
| HR (95%CI)[b] | | Reference | 1.87 (0.72-4.85) | 1.82 (1.16-2.87) | 1.31 (0.74-2.34) | 0.219 |
| | | | | | | |
| **Non-cardia** | | | | | | |
| No. participants | 94232 | 39742 | 4131 | 31796 | 18442 | |
| GC cases | 451 | 45 | 36 | 315 | 55 | |
| HR (95%CI)[b] | | Reference | 7.13 (4.53-11.23) | 4.97 (3.57-6.12) | 3.45 (2.30-5.16) | <0.001 |
| | | | | | | |
| **Unspecified / Overlapping** | | | | | | |
| No. participants | 94232 | 39742 | 4131 | 31796 | 18442 | |
| GC cases | 144 | 21 | 7 | 94 | 22 | |
| HR (95%CI)[b] | | Reference | 3.14 (1.30-7.59) | 3.43 (2.06-5.72) | 2.89 (1.56-5.36) | 0.498 |

Abbreviations: GC, gastric cancer

[a] NHW is defined as those who were born in the US and self-identified as non-Hispanic White, regardless of where their parents were born.

[b] Adjusted for age, sex, family history, sodium and fruit consumption, alcohol intake, education, body mass index, history of diabetes, ulcers, polyps, smoking

Supplementary Table 5. Sensitivity analysis of gastric cancer risk by generation immigration status among Japanese Americans, with the reference group limited to White individuals born in the US with both parents born in the US or Europe

| Sensitivity analysis criteria | Total Population | NHW | First generation | Second generation, Both parent Japan-born | Second generation, one parent Japan-born | Third generation | p-trend |
|---|---|---|---|---|---|---|---|
| **White ethnicity by definition, as the reference group[a,b]** | | | | | | | |
| No. participants | 92218 | 37765 | 4088 | 23241 | 8615 | 18461 | |
| GC cases | 705 | 92 | 48 | 370 | 99 | 96 | |
| HR (95%CI) | | Reference | 4.91 (3.42-7.05) | 3.89 (3.04-4.98) | 3.60 (2.69-4.82) | 2.70 (2.01-3.63) | <0.0001 |
| | | | | | | | |
| **Excluding the first two years of follow-up[b]** | | | | | | | |
| No. participants | 92726 | 39007 | 4110 | 22770 | 8506 | 18333 | |
| GC cases | 650 | 93 | 47 | 334 | 89 | 87 | |
| HR (95%CI) | | Reference | 5.14 (3.57-7.40) | 3.57 (2.78-4.58) | 3.40 (2.52-4.59) | 2.74 (2.03-3.71) | <0.0001 |
| | | | | | | | |
| **Excluding the first five years of follow-up[b]** | | | | | | | |
| No. participants | 89720 | 37555 | 3992 | 21519 | 8213 | 17979 | |
| GC cases | 539 | 77 | 36 | 276 | 75 | 75 | |
| HR (95%CI) | | Reference | 4.85 (3.21-7.31) | 3.37 (2.57-4.43) | 3.46 (2.49-4.80) | 3.12 (2.25-4.34) | <0.0001 |
| | | | | | | | |
| **Including those who exclusively reported Japanese ethnic or racial background[b]** | | | | | | | |
| No. participants | 91763 | 39779 | 4096 | 23221 | 8440 | 16227 | |
| GC cases | 701 | 103 | 48 | 370 | 96 | 84 | |
| HR (95%CI) | | Reference | 4.69 (3.29-6.69) | 3.70 (2.91-4.69) | 3.37 (2.53-4.48) | 2.54 (1.89-3.43) | <0.0001 |

[a] NHW is defined as those who were born in the US and self-identified as White, whose parents were born either in the US or Europe

[b] Model adjusted for age, sex, fruit consumption, alcohol intake, education, body mass index, history of diabetes, ulcers, polyps, family history, smoking, sodium consumption.
